# The Immunotherapy and Immunosuppressive Signaling in Therapy-Resistant Prostate Cancer

**DOI:** 10.3390/biomedicines10081778

**Published:** 2022-07-22

**Authors:** Pengfei Xu, Logan J. Wasielewski, Joy C. Yang, Demin Cai, Christopher P. Evans, William J. Murphy, Chengfei Liu

**Affiliations:** 1 Department of Urologic Surgery, University of California Davis, Sacramento, CA 95817, USA; pfxu@ucdavis.edu (P.X.); ljwasielewski@ucdavis.edu (L.J.W.); jcyang@ucdavis.edu (J.C.Y.); cpevans@ucdavis.edu (C.P.E.); 2 College of Animal Science and Technology, Yangzhou University, Yangzhou 225009, China; demincai@yzu.edu.cn; 3 UC Davis Comprehensive Cancer Center, University of California Davis, Sacramento, CA 95817, USA; wmjmurphy@ucdavis.edu; 4 Department of Internal Medicine, University of California Davis, Sacramento, CA 95817, USA

**Keywords:** prostate cancer, immunotherapy, immunosuppressive signaling, tumor immune microenvironment, checkpoint inhibitor, therapy resistance

## Abstract

Prostate cancer is one of the most common malignant tumors in men. Initially, it is androgen-dependent, but it eventually develops into castration-resistant prostate cancer (CRPC), which is incurable with current androgen receptor signaling target therapy and chemotherapy. Immunotherapy, specifically with immune checkpoint inhibitors, has brought hope for the treatment of this type of prostate cancer. Approaches such as vaccines, adoptive chimeric antigen receptor-T (CAR-T) cells, and immune checkpoint inhibitors have been employed to activate innate and adaptive immune responses to treat prostate cancer, but with limited success. Only Sipuleucel-T and the immune checkpoint inhibitor pembrolizumab are approved by the US FDA for the treatment of limited prostate cancer patients. Prostate cancer has a complex tumor microenvironment (TME) in which various immunosuppressive molecules and mechanisms coexist and interact. Additionally, prostate cancer is considered a “cold” tumor with low levels of tumor mutational burden, low amounts of antigen-presenting and cytotoxic T-cell activation, and high levels of immunosuppressive molecules including cytokines/chemokines. Thus, understanding the mechanisms of immunosuppressive signaling activation and immune evasion will help develop more effective treatments for prostate cancer. The purpose of this review is to summarize emerging advances in prostate cancer immunotherapy, with a particular focus on the molecular mechanisms that lead to immune evasion in prostate cancer. At the same time, we also highlight some potential therapeutic targets to provide a theoretical basis for the treatment of prostate cancer.

## 1. Introduction

Prostate cancer has become the second most common cancer and the fifth most deadly cancer in the world [1]. In the United States, prostate cancer is the second leading cause of cancer-related death in men, projected to have 268,490 new cases and 34,500 deaths in 2022 [2]. Although localized prostate cancer may be successfully treated with radical prostatectomy or radiotherapy [3], standard androgen deprivation therapy (ADT) followed by chemotherapy (Taxanes), androgen synthesis inhibitor (abiraterone), or androgen receptor (AR) antagonists (enzalutamide, apalutamide, and darolutamide) remains the primary treatment option for patients with advanced prostate cancer [4]. Unfortunately, despite initial response to the current therapy, all patients eventually will develop from castration-resistant prostate cancer (CRPC) to drug-resistant CRPC through androgen-dependent or androgen-independent mechanisms. Androgen-dependent mechanisms include a rise in AR splice variants [5,6], AR overexpression [7], intratumoral or alternative androgen biosynthesis [8], and AR mutations [9], while androgen-independent mechanisms are mediated through the activation of DNA repair pathways [10], PI3K/AKT/mTOR pathways [11,12], BRAF-MAPK [13], Wnt signaling pathways [14,15], glucocorticoid receptor pathways [16], and neuroendocrine differentiation [17]. Multiple clinical trials are exploring therapeutic strategies to overcome these resistance mechanisms, including those utilizing immunotherapies that work in conjunction with the patient’s immune system.

Harnessing the patient’s immune system to fight tumors has reinvigorated the field of cancer therapy over the past few decades. Tumor immunotherapy has ushered in a new era of cancer treatment, bringing hope to patients with advanced stages of cancers, including prostate cancer [18]. In fact, immune checkpoint inhibitors targeting programmed death 1 (PD-1)/programmed death ligand 1 (PD-L1) and CTLA-4 (both of which suppress T-cell proliferation) have shown unprecedented, sustained responses in certain tumor types. These cancers (e.g., melanoma [19], kidney cancer [20], and non-small-cell lung cancer [21]) are often referred to as having “hot tumors” because of the considerable reliance they have on immune checkpoints for propagation. In contrast, “cold tumors”, such as prostate cancer, have low tumor immunogenicity [22] and an active immunosuppressive tumor microenvironment (TME) [23], resulting in a very limited response to immunotherapy. The majority of prostate tumors are identified as immune-ignorant, typically characterized by decreased levels of antigen-expressing molecules [24], the low expression of genes involved in antigen processing and presentation, and deficient cytotoxic T-cell recruitment and activation [25,26]. These factors contribute to prostate tumors being “cold”, since they are less responsive to immune factors as other cancers. This leads to difficulties when testing immunotherapies, such as immune checkpoint inhibitors in prostate cancer. A better understanding of the molecular mechanisms that lead to immunosuppression in prostate cancer and the development of better treatment options will help address the current clinical dilemma in prostate cancer immunotherapy.

The purpose of this review is to summarize emerging developments in prostate cancer immunotherapy, with a particular focus on the molecular mechanisms that constitute immune evasion in prostate cancer. Moreover, we also compile potential therapeutic targets to provide a theoretical basis for prostate cancer immunotherapy.

## 2. The Development of Prostate Cancer Immunotherapy

### 2.1. Sipuleucel-T

Sipuleucel-T is an active cellular immunotherapy that stimulates T-cell immune responses and is currently the only anticancer vaccine approved for the treatment of asymptomatic or mildly symptomatic metastatic CRPC (mCRPC) patients. It consists of autologous peripheral blood mononuclear cells (PBMCs) that are activated in vitro with a recombinant fusion protein (PA2024) containing the prostate antigen, prostatic acid phosphatase, and granulocyte-macrophage colony stimulating factor (GM-CSF) to expand antigen-loaded antigen-presenting cells (APCs) and then reinfuse back to patients after 36–44 h of in vitro culturing to sensitize/activate the patient’s own T cells [27]. Compared with a placebo, patients in the Sipuleucel-T arm had a median survival of 4.1–4.5 months longer [27,28] and a 22–33% lower risk of death [29]. In addition, preliminary safety and efficacy data for the combination of Sipuleucel-T with AR-targeting agents [30], PD-L1 inhibitor atezolizumab [31], or Radium-223 [32] in mCRPC patients are still in development. Further studies in larger trials are necessary to verify the positive effects of these combination therapies. Sipuleucel-T is a landmark of prostate cancer immunotherapy. However, the modest efficacy and economically low cost-effectiveness restrict its application. Several other prostate cancer vaccines, including PROSTVAC/PCa and Anti-RhoC, have been tested in clinical trials (Table 1).

### 2.2. Adoptive Immune Cell Immunotherapy

Adoptive cell therapy, which utilizes autologous activated immune effector cells to eradicate tumor cells, has achieved promising results in a variety of hematological malignancies [44]. However, as they lack specific tumor antigens and due to the existence of an immunosuppressive TME in solid tumors and the expression of inhibitory molecules (such as PD-1) by tumor cells, the activity and proliferation of T cells are always suppressed. Additionally, the abilities of T cells to recognize and kill tumor cells are impaired, resulting in the limited application of chimeric antigen receptor-T (CAR-T) in solid tumors [45]. In prostate cancer, autologous T lymphocytes collected by peripheral blood leukapheresis can be genetically engineered to express CARs that recognize specific prostate tumor antigens and obtain specific immune effector cells. CAR-T cells identify and infiltrate tumor cells by targeting surface antigens without the presence of APCs. There have been five generations of CARs, and their molecular structures and physical properties significantly alter CAR-T cells’ effects in therapeutics [46]. Currently, PSMA-CAR-T cells (NCT04429451 and NCT04249947), PSCA-CAR-T cells (NCT03873805), and CAR-T-PSMA-TGFβRDN69 cells (NCT03089203 and NCT04227275) are highly effective in mCRPC tested in phase I or phase II clinical trials [37,47,48,49,50]. Natural killer (NK) cells can also be engineered to express the CAR, and recruitment is currently ongoing for an early phase I clinical trial evaluating anti-PSMA CAR-NK cells (NCT03692663) [51].

### 2.3. Immune Checkpoint Inhibitors

The advent of immune checkpoint inhibitors targeting the PD-1/PD-L1 pathway has reinvigorated the treatment of various advanced cancers [52]. However, the stronger immunosuppressive microenvironment renders prostate cancer to be less susceptible to immune checkpoint blockades. Studies have illustrated that PD-1/PD-L1 inhibitors provide suboptimal clinical benefit in an unselected population of patients with mCRPC [38,53]. Nonetheless, long-term survival benefits and sustained complete responses have been reported in some mCRPC patients receiving a CTLA-4 inhibitor (Ipilimumab) [54], suggesting that selected prostate cancer patients may benefit from a checkpoint blockade. Accordingly, a more widely accepted explanation for the suboptimal clinical activity is that the majority of mCRPC patients have an immunosuppressive TME with the low infiltration of CD8+ T cells and an increased influx of immunosuppressive cells. Therefore, clinical trials designed for combining anti-PD-1/PD-L1 agents with other anticancer treatments, including inhibitors of different immune checkpoint pathways or other systemic anticancer treatments, mostly failed in prostate cancer at the current stage (Table 1).

In a phase Ia study using atezolizumab in patients with mCRPC which progressed after Sipuleucel-T or enzalutamide treatment, 8.6% of patients had a 50% PSA response, and only one patient had an objective partial response, with a median overall survival (OS) of 14.7 months [38]. In the phase II KEYNOTE-199 trial, pembrolizumab had less than 10% of the PSA response rate, less than 5% of an overall response rate (ORR), and 2.1, 2.1, and 3.7 months of radiographic PFS (rPFS) for the three cohorts, respectively (Cohort 1: PD-L1-positive; Cohort 2: PD-L1-negative; Cohort 3: bone-predominant disease, regardless of PD-L1 expression) [40]. In addition, a phase III trial (IMbassador250) showed that the addition of the PD-L1 inhibitor atezolizumab did not increase the efficacy of enzalutamide in mCRPC patients whose disease had progressed on abiraterone (*n*  =  759) [39]. The OS endpoints were not reached, and the trial was terminated. From the trial, longer PFS was associated with the addition of atezolizumab to enzalutamide in men selected for PD-L1 immune checkpoint expression and high levels of CD8+ T cells. Emerging data suggested that AR is a negative regulator of CD8+ T cells in responding to anti-PD1/PD-L1 treatment [55]. AR status in T cells may act as an important efficacy predictor to the immune checkpoint inhibitors, and complete androgen axis blockage is critical to achieve the best antitumor results by immune checkpoint inhibitors in CRPC patients. Thus, the potential synergy between atezolizumab and enzalutamide might be useful in certain selected patients and should be verified by future clinical trials [56].

Ipilimumab, a popular inhibitor of the immune checkpoint molecule CTLA-4, was used to treat in prostate cancer patients after it was approved by the FDA to treat melanoma. Ipilimumab, when taken as a monotherapy or combination treatment in phase I/II clinical trials of mCRPC, did not exacerbate immune-related adverse effects [57]. The combination of ipilimumab and radiotherapy improved OS (2- to 3-fold higher) in patients with mCRPC after docetaxel, while combination therapy with the PD-1 inhibitor nivolumab showed only modest activity in AR-V7-positive mCRPC patients [41]. The ongoing phase II trial of nivolumab in combination with ipilimumab (CheckMate 650) divides patients with mCRPC before and after chemotherapy into two cohorts. A preliminary analysis on 90 treated patients showed objective response rates of 25% and 10%, respectively, with a median OS of 19 and 15.2 months before and after chemotherapy; two patients in each cohort had complete responses [42].

Despite the unfavorable results in prostate cancer by current immune checkpoint inhibitors, PD-1 inhibitors including nivolumab and pembrolizumab, anti-PD-L1 inhibitors including atezolizumab and avelumab, and anti-CTLA4 inhibitor ipilimumab [58] continue to be rigorously tested in multiple clinical trials in CRPC patients (Table 2). The ongoing clinical trials for immune checkpoint inhibitors in prostate cancer will further examine the applicability of these inhibitors to clarify whether they may improve treatment outcomes for patients with CRPC either alone or in combination with other treatment strategies.

## 3. Role of TME in Therapy-Resistant Prostate Cancer Immune Evasion

**Figure 1:**
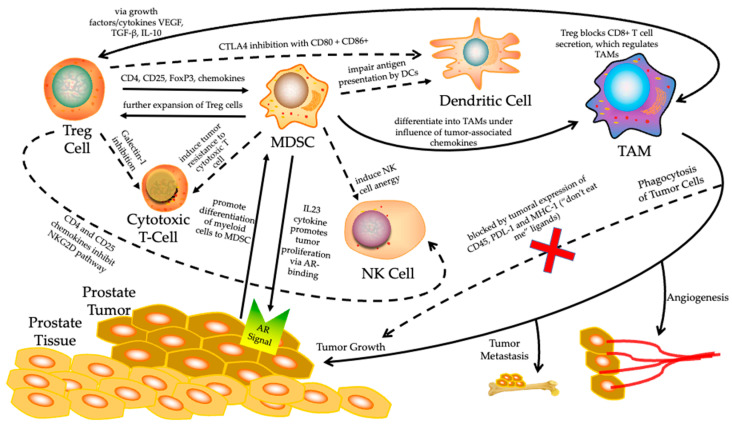
The tumor immune microenvironment (TIME) surrounding prostate cancer. It is known to be highly immunosuppressive, utilizing many cellular pathways which inhibit normal immune function and promote a tumor’s unchecked proliferation. Solid arrows represent upregulation of that cell’s expression or a specific function at the end of the arrow. Dashed arrows indicate inhibition of the cell or function at the end of the arrow. Specific mechanisms by which upregulation or inhibition work are summarized on their respective arrows. Treg, regulatory T; MDSC, myeloid-derived suppressor cell; NK, natural killer; TAM, tumor-associated macrophage; DC, dendritic cell; AR, androgen receptor.

Prostate cancer is considered a heterogeneous disease with a highly complex TME. Encompassed by a non-inflammatory TME and the low expression of neoantigens which would signal unchecked cell growth, prostate tumors have difficulty being differentiated by the immune effector system. Meanwhile, prostate tumors tend to evade antitumor immune cells through the secretion of immunosuppressive factors such as interleukin 1 beta (IL-1β), IL-10, and TGF-β in the TME and thereby induce the differentiation of myeloid cells into myeloid-derived suppressor cells (MDSCs). Concurrently, the increased infiltration of regulatory T cells (Tregs) and tumor-associated macrophages, in combination with decreased circulating NK cells in prostate cancer tissues, are associated with worse prognosis and resistance to immunotherapy [59] (Figure 1).

### 3.1. Regulation of Tumor Immune Microenvironment (TIME)

In order to promote tumor growth, the TIME consists of tumors and their surrounding blood vessels, extracellular matrixes (ECMs), fibroblasts, immune cells, bone marrow-derived inflammatory cells, various signaling molecules, and other components, which play crucial roles in the antitumor immune response [60]. T-cell composition, vascular networks, and cytokine diversity in the TIME are determinants of T-cell-mediated antitumor responses. There is complex interplay between the stromal cell background of fibroblast infiltration, the metabolic state promoted by the disturbed vasculature, and the subsequent hypoxia leading to an immunosuppressive TIME [61]. Cytokines such as VEGF, TGF-β, and IL-10 are responsible for the recruitment of Tregs in the TIME and inhibit the proliferation, activation, and infiltration of cytotoxic lymphocytes. Therefore, the TIME plays a key role in prostate cancer progression and immune evasion. Previous studies suggest that the presence of tumor-infiltrating lymphocytes (TILs) is generally associated with better prognosis [62,63]. However, prostate cancer TIL populations are mainly composed of CD4+FOXP3+CD25+ Treg cells and M2-type tumor-associated macrophage (TAM) cells, which contribute to the production of inhibitory cytokines and the maintenance of self-tolerance to suppress the immune response [64]. They are associated with a higher risk of metastatic disease at diagnosis and worse distant metastasis-free survival [65]. In addition, chemokines (such as CCL22) and chemokine receptors (such as CXCR4 and CXCR5) expressed by Tregs and MDSCs also contribute to immunosuppressive TIME formation in prostate cancer [66].

### 3.2. The Role of MDSC in Prostate Cancer Immune Evasion

The inhibition of antitumor immune responses is one of the main mechanisms by which tumor cells evade destruction by the immune system. MDSCs represent the major immunosuppressive cells present in the TIME that sustain cancer progression. MDSCs were first characterized in the early 1970s as a grouping of cells that naturally suppress cytotoxic T-cell activity and function and are phenotypically similar to monocytes and neutrophils, but not to B and T lymphocytes, NK cells, and macrophages [67]. It is now known that MDSCs are a highly heterogeneous category of cells originating in the bone marrow, and these dedifferentiated cells can suppress antitumor immune activity and promote immune evasion. They were identified as a population of immature granulocytes (G-MDSC) and a population of monocyte morphology (M-MDSC) [68]. Their immunosuppressive properties include the inhibition of T-cell activation and dendritic cell maturation, the induction of NK cell anergy, and the promotion of the de novo expansion of Tregs [69]. In addition, they can differentiate into fibroblasts, endothelial cells, osteoclasts, and TAMs when influenced by tumor-cell-based chemokines, which play a key role in tumor invasion, angiogenesis, and metastasis [70]. The accumulation of MDSCs in the TIME has been associated with disease progression and poor prognosis in a variety of tumors [71]. Thus, MDSCs are a major hurdle for many cancer immunotherapies, and their targeting may be a beneficial strategy to improve the efficiency of immunotherapeutic interventions.

MDSCs may represent novel prognostic biomarkers due to the marked accumulation of various subtypes found in the peripheral blood of prostate cancer patients compared with healthy age-matched donors [72]. Importantly, MDSC levels were associated with PSA levels, disease burden, and clinical outcomes after different treatments in prostate cancer patients [73]. Notably, patients with high levels of G-MDSC before treatment had a shorter median OS. In a Pten null (Pb-Cre+; Pten^lox/lox^) murine prostate cancer model, PTEN loss promotes the expansion of MDSCs in hematopoietic tissues [74]. Recent reports have shown the IL23 cytokine secreted by MDSCs can activate AR signaling in prostate tumor cells to promote cell proliferation and survival. Consequently, blocking IL23 restores androgen therapy sensitivity [75]. In addition, the TLR9-STAT3-Arginase-1 signaling pathway and the nitration of lymphocyte-specific protein tyrosine kinase (LCK) cells were also reported to be involved in the functional regulation of MDSCs in CRPC [76,77]. The depletion of MDSCs, impairment of MDSC function and recruitment, as well as the promotion of their differentiation and maturation will help to enhance antitumor immune responses and improve therapeutic intervention in CRPC [78].

### 3.3. The Treg Cells in Prostate Cancer Immune Evasion

As a predominant immunosuppressive cell type in the TME, Treg cells exert immunosuppressive effects through a variety of different mechanisms. They directly suppress the immune response through the secretion of immunosuppressive cytokines (TGF-β and IL-10) [79]. They can induce immune cell death by secreting perforin/granzyme B, etc. They can also inhibit the growth and proliferation of T cells and induce T-cell apoptosis through Galectin-1 [80]. In addition, CTLA-4 expressed on Tregs can bind with high affinity to CD80 and CD86 on dendritic cells (DCs) and regulate APCs function, thereby inhibiting T-cell function [81]. They can also promote tumor angiogenesis, migration, metastasis and drug resistance [82]. In the prostate cancer microenvironment, Treg cells downregulate NK cells and cytotoxic lymphocytes (CTLs), while supporting MDSCs and M2 macrophages [83]. Therefore, they play an important role in the progression of prostate cancer. Currently, targeting Treg cells is a possible therapeutic strategy for the treatment of prostate cancer patients. The combination of anti-CTLA-4 antibody and anti-CD25 antibody can significantly inhibit tumor growth and progression in vivo [84]. Targeting Tregs with anti-CCR4 antibodies can significantly reduce Treg numbers [85]. Entinostat, a histone deacetylase inhibitor, inhibits the proliferation and growth of Treg populations by activating STAT3 acetylation and reducing FoxP3 expression, suggesting a novel and effective approach to modulate the immune system and treat prostate cancer [86]. Finding effective strategies to target Treg cells can serve as a major area of research for future studies evaluating prostate cancer therapy.

## 4. The Molecular Mechanisms of Immunosuppressive Signaling Activation in Prostate Cancer

**Figure 2:**
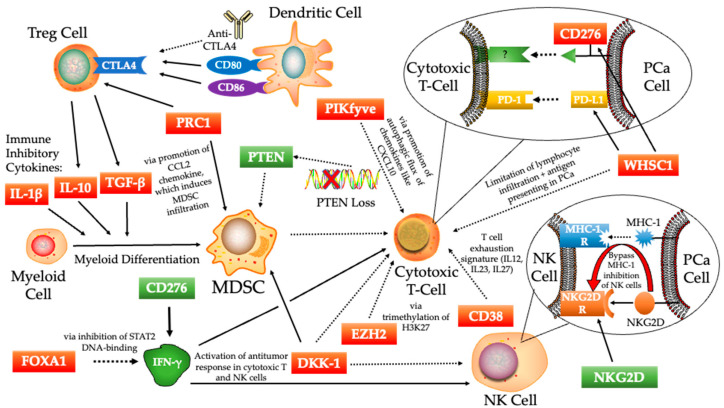
Specific mechanisms for different molecules in the tumor immune microenvironment (TIME) of prostate cancer. These are novel targets of interest which have been highlighted through publications in recent years. Solid arrows represent upregulation of that cell’s expression or a specific function at the end of the arrow. Dashed arrows indicate inhibition of the cell or function at the end of the arrow. The color of the boxed molecules represents whether they are immunosuppressive (red) or an immune enhancer (green). Note that CD276 had co-stimulatory and inhibitory roles in the immune response. T-reg, regulatory T; MDSC, myeloid-derived suppressor cell; NK, natural killer; IFN-γ, interferon-gamma; PCa, prostate cancer.

While the role and function of specific immune cells in the prostate TME are well-researched, the plethora of mechanisms behind their activation, inhibition, and recruitment have not been well-studied on a molecular level until recently. To investigate this, researchers have discovered several genes involved in immunosuppressive signaling activation in prostate cancer. These specific proteins and molecules have been further studied to elucidate the mechanisms by examining the effects of their overexpression or inhibition on the ratio of immune cells. Here, we discuss these molecules, as well as the proposed mechanisms by which they work to inhibit or enhance the immune system’s response to prostate tumors with a summarized illustration in Figure 2.

### 4.1. CD276 (B7-H3)

The upregulation of inhibitory B7 molecules in the TME is highly correlated with tumor immune evasion [87]. Recent studies have shown that a new member of the B7 family (CD276, B7-H3) is overexpressed in a variety of cancer cells and is associated with disease progression, suggesting that it may serve as a potential new target for antitumor therapy [88,89]. CD276 is believed to have conflicting co-stimulatory and co-suppressive roles in immune responses. As a co-stimulatory molecule, it assists in T-cell activation and interferon gamma (IFN-γ) production [90], and conversely, it also inhibits the proliferation of CD4+ and CD8+ T cells [91]. However, in the process of tumor development, CD276 can promote tumor invasion and metastasis [92,93,94] and contributes to the resistance of anticancer drugs through various mechanisms [95,96]. In prostate cancer, CD276 is a useful biomarker to identify highly aggressive metastatic prostate cancer. The high expression of CD276 was more common in patients with metastatic prostate cancer (31%) than with localized cancer (12%). In patients with localized cancer, CD276 expression status was not associated with biochemical recurrence-free survival. However, in patients with metastatic cancer, high CD276 expression was significantly associated with high disease-specific and overall mortality [97]. Moreover, CD276 is highly expressed in advanced prostate cancer and correlates with the altered loss of BRCA2 and ATM function, as well as low intratumoral TILs. Therefore, CD276 may be an actionable target for the treatment of this subgroup of prostate cancer [98].

### 4.2. PTEN

PTEN is a tumor suppressor gene with dual-specific phosphatase activity, which plays an important role in cell growth, apoptosis, adhesion, migration, and infiltration. The incidence of PTEN gene mutation in primary prostate cancer and metastatic prostate cancer is 5–27% and 30–60%, respectively. About 50% of prostate cancer patients have heterozygous loss and 10% homozygous loss of PTEN [99]. The loss of the PTEN protein is related to high Gleason and pathological grades [100], affects AR signaling and cell sensitivity to ADT [12], and regulates PI3K/AKT/mTOR signaling pathway [101]. In addition to its function on prostate cancer biology, PTEN affects immune cell composition in the TME. Studies have shown that PTEN deficiency is associated with the immunosuppressive state of prostate cancer, including lower CD8+ T cell and higher FoxP3+ Treg cell abundance, while the lower abundance of M2 macrophages was found in PTEN-deficient metastatic lymph nodes [102]. Pre-clinical studies have shown that PTEN deficiency leads to an increase in the number of tumor-infiltrated MDSCs in the TME [74]. In addition, PTEN deficiency inhibits innate and adaptive immune responses by impairing the activation of type I IFN and NF-κB pathways [103], suppressing the antigen-presenting function of DCs, and the recruitment and activation of T and NK cells [104,105]. PTEN loss was also associated with the increased expression of the immunosuppressive molecules IDO1 and CD276 in prostate cancer tissue [102,105,106]. At the same time, the loss of PTEN was associated with the increased expression of a series of chemokines (CXCL12 and CXCL8), which indirectly caused the formation of an immunosuppressive TME in prostate cancer [107,108]. Therefore, discovering how to restore PTEN function through direct or indirect ways and develop novel PI3K-AKT-mTOR inhibitors with stronger potency and selectivity will give hope to those CRPC patients with PTEN loss.

### 4.3. FOXA1

Forkhead-box A1 (FOXA1) encodes a precursor factor that interacts with dense chromatin independently of other proteins and can directly regulate chromatin structure [109]. This usually involves the “opening” of chromatin, but under specific circumstances, FOXA1 can also recruit other factors, such as transducin-like enhancer (TLE) proteins, to promote chromatin inaccessibility [110]. FOXA1 has been identified as a promoter of prostate cancer pathogenesis and progression [111,112,113,114]. In addition to its chromatin modulation function, FOXA1 may also regulate the TME. Inflammatory response genes have been found to be upregulated in the tumors of prostate cancer patients with low FOXA1 expression. FOXA1 directly inhibited hypoxia-inducible factor 1-α (HIF1A) expression by binding to its enhancer, while FOXA1 depletion enhanced the expression of HIF1A and monocyte chemoattractant protein-1 (MCP-1/CCL2). This led to immunosuppression and promoted the infiltration of M2-type macrophages. Moreover, the inhibition of this HIF1A-CCL2 axis with HIF1A inhibitors or CCL2 antibodies blocked macrophage infiltration [115]. The overexpression of FOXA1 may play a key role in prostate cancer immune coldness by regulating IFN-responsive gene expression. The overexpression of FOXA1 was inversely correlated with IFN signaling activity and antigen-presenting gene expression in prostate cancer patients, contributing to immune escape and conferring resistance to immune checkpoint inhibitors [116]. Therefore, FOXA1 may serve as a prognostic factor predicting treatment resistance and be a viable target for sensitivity to immunity and chemotherapy.

### 4.4. EZH2

EZH2 is the methyltransferase catalytic subunit of polycomb repressive complex 2 (PRC2) that catalyzes the trimethylation of histone H3K27 to repress gene transcription [117]. EZH2 can negatively regulate IFN-inducible genes, immune checkpoint molecules, and major histocompatibility complex (MHC) expression [118]. It is also an important regulator of CD4+T-cell and Treg cell differentiation and plays a key role in immune regulation. EZH2 has been shown to be a key mediator of acquired prostate tumor immune evasion, and its enhanced function often correlates with an immunosuppressive TME, immunotherapy resistance, and the inhibition of T-cell differentiation and infiltration. Studies have shown that increased EZH2 expression and activity are key events in prostate cancer initiation and progression [119]. The inhibition of EZH2 upregulates the induction of IFN-stimulated genes in prostate cancer and promotes a marked increase in CD4+ and CD8+ T cells in the TME, reversing the anti-PD-1 therapy resistance of B6-HiMYC PCa transgenic tissue transplant model [120].

### 4.5. DKK-1

Dickkopf-1 (DKK-1), a member of the Dickkopf family of bone factors, inhibits bone formation by blocking the canonical Wnt signaling pathway and directly promotes angiogenesis through VEGFR2. Elevated levels of DKK-1 in a variety of tumors are associated with poor prognosis, suggesting that DKK-1 represents a common malignant tumor biomarker [121]. Recent studies have confirmed that DKK-1 also plays an important role in inflammatory and immune processes [122]. Neutralizing anti-DKK-1 antibodies helps to attenuate MDSC accumulation in the tumor microenvironment and restore T-cell numbers. In contrast, knocking out β-catenin in myeloid cells abolished the effect of anti-DKK-1 antibodies on tumor growth [123]. These results suggest that DKK-1 elicits immunosuppressive effects that indirectly promote tumor growth. Accumulating data suggest that the relative increase in DKK-1 expression in prostate cancer may have direct effects on tumor proliferation and the cell cycle [124], while the inhibition of DKK-1 reduces the tumor burden in prostate cancer [125]. Additionally, high DKK-1 serum levels at diagnosis were associated with significantly shorter overall and disease-specific survival. Multivariate analysis defined high serum DKK-1 levels as an independent prognostic marker for prostate cancer [126]. The analysis of prostate cancer immune cells revealed that DKK1 expression levels correlated with features of immunosuppression, including increased M2 macrophages, decreased CD8+ T cells, and lower levels of activated NK cells [127]. In human PCa models (PC3), DKK1 blockades slowed tumor growth in an NK-cell-dependent manner [128]. A phase 1b/IIa parallel-arm study of the DKK1 inhibitor DKN-01 as monotherapy or in combination with docetaxel in advanced prostate cancer with elevated DKK-1 is ongoing (NCT03837353).

### 4.6. WHSC1

The Wolf–Hirschhorn syndrome candidate 1 (WHSC1, also known as MMSET and NSD2) protein gene encodes a SET-domain-containing histone methyltransferase that targets H3K36me2 [129]. It promotes tumor growth and metastasis, and its elevated expression is associated with poor prognosis. Although tumoral progression under WHSC1 is well-described [130,131], its role as an epigenetic modifier in the communication between prostate cancer and the immune system remains poorly explored. Recent studies have shown that elevated WHSC1 expression is positively correlated with the presence of an immunosuppressive microenvironment. It limits lymphocyte infiltration in prostate tumors and reduces antigen processing and presentation, and it also inhibits local activation of immune pathways [132]. In a mouse model of prostate cancer (TRAMP C-2), the pharmacological inhibition of WHSC1 reduced the infiltration of CD4+CD25+ Tregs and upregulated MHC-II expression on CD45+CD11c+ DCs. It enhanced the antitumor functional activity of infiltrating immune cells and restricted the immunosuppressive transcriptional program from M2 macrophages [133]. In addition, WHSC1 shapes the epigenetic landscape of prostate cancer cells by altering DNA methylation and chromatin accessibility. WHSC1 has been shown to alter ubiquitinase and proteasome genes, helping to generate new antigenic peptides. When WHSC1 was inhibited, the expression of immune and MHC genes was upregulated, indicating that the processed peptides could reach the cell surface and be recognized by the immune system. It also controls the expression of CD276 and PD-L1. This suggests antitumor immune responses to the TME may be limited with WHSC1 through distinct yet complementary mechanisms [132]. These studies reveal the pharmacological inhibition of WHSC1 has the potential to serve as an effective adjuvant for future use as immunotherapy in prostate cancer.

### 4.7. NKG2D

NKG2D, an activating receptor expressed on the surface of NK cells and CD56+ and CD8+ T cells, plays a critical role in the innate immune system and is involved in the recognition and killing of virus-infected cells and tumor cells by NK cells [134]. NK cells are a preliminary factor for immune surveillance and tumor growth inhibition in the body’s immune system and can terminate tumor cells without antigen stimulation [135]. NK cells express activating and inhibitory receptors on their surface, and when the signal triggered by the activating receptor exceeds the signal triggered by the inhibitory receptor, the NK cell produces effector functions [136]. MHC-I expression on the surface of tumor cells is diminished, so they cannot be recognized by inhibitory receptors and are unable to transmit negative regulatory signals. This results in NK cells which present in an activated state to kill tumor cells. However, even though they may express MHC-I, some tumor cells can still be recognized by NK cells due to the co-expression of NKG2D ligands, which activates NK cells’ “missing-self” function and allows tumor cells to escape from immune surveillance [137]. Therefore, NK cells have recently been at the forefront of many immunotherapy strategies, and several immunotherapeutic approaches targeting the NKG2D-NKG2DL axis are being developed to remodel the TME and unleash the antitumor effects of NK cells and cytotoxic CD8+ T cells.

In prostate cancer patients, there is ample evidence that NK cell dysfunction is associated with poor clinical outcomes across disease stages [138]. The blood levels of the NK-activating receptor (NKp46) were decreased in prostate cancer patients and negatively correlated with PSA levels [139]. Prostate cancer cells secrete exosomes containing the surface NKG2D ligands MICA/B and ULBP-2, which selectively downregulate the expression of NKG2D on NK cells, thereby negatively regulating the cytotoxic function of NK cells [140]. These tumor cells induce the expression of inhibitory receptors (ILT2/LILRB1) and downregulate the expression of NK-cell-activating receptors NKp46, NKG2D and CD16, thereby preventing recognition and killing via NK cells. In addition, the increased secretion of the inhibitory cytokine TGFβ in the TME can also significantly inhibit NK cell function [139]. Numerous reviews have addressed NK cells as potential targets for immunotherapy [135,141]. Therefore, eliminating tumors by restoring NK cell activation and effector function, thus converting “cold” tumors into “hot” tumors, is a promising therapeutic approach [142].

### 4.8. CD38

CD38, which belongs to the ADP-ribosyl cyclase family, is widely expressed on the surface of non-hematopoietic cells and immune cells. It plays different roles in lymphocyte development, activation, and differentiation, and promotes atypical glandular growth by catalyzing the conversion of nicotinamide adenine dinucleotide (NAD+) to ADP-ribose (ADPR) and cyclic ADPR glycoside synthesis [143,144]. This will lead to an augmentation of adenosine that depresses antitumor immunity through its direct effects on multiple immune cell subsets [145]. There is increasing evidence that CD38 is involved in tumor immune evasion; in particular, CD38 mRNA expression in mCRPC correlates with IL12, IL23, and IL27 signaling signatures as well as immunosuppressive adenosine signaling and T-cell exhaustion signatures. Additionally, CD38+ tumor-infiltrating immune cell density has been shown to increase significantly in mCRPC tumors and correlate with poorer OS [145]. Since NAD+ is essential for the regulation of enzymatic processes of cellular metabolism and lymphocyte differentiation and function, CD38 may also affect TILs function by depleting NAD+, while CD38 inhibition leads to the metabolic reprogramming of T cells. Strategies targeting this CD38/NAD+ axis may improve the efficacy of antitumor-adoptive T-cell therapy [146]. In a clinical trial (NCT03367819), the combination of anti-CD38 (isatuximab) and PD-1 (cemiplimab) monoclonal antibodies in patients with mCRPC resulted in a median reduction of CD38+ tumor-infiltrating immune cells from 40% to 3% and the activation of peripheral T cells [147]. However, the sample size still needs to be expanded in further clinical trials to verify the results.

### 4.9. PRC1

Polycomb repressive complex 1 (PRC1), composed of four subunits PHC, BMI-1, CBX, and Ring 1A/B, is an E3 ubiquitin ligase that monoubiquitinates histone H2A lysine 119 (H2AK119ub1), which silences target gene expression and promotes dedifferentiation and stemness during development and cancer [148]. Studies have shown that PRC1 promotes the recruitment of MDSCs, TAMs, and Tregs to the TME, thereby creating a highly immunosuppressive microenvironment in double-negative prostate cancer (DNPC). In contrast, the inhibition of PRC1 reversed immunosuppression at bone metastases and suppressed angiogenesis in a DNPC model (Pten ^pc−/−^ Smad4 ^pc−/−^) [149]. Furthermore, some canonical PRC1 subunits or key components are also closely related to the malignant biological behavior of prostate cancer. For example, Bmi1 is considered to be a marker of CRPC luminal stem cells [150]. Additionally, CBX2 has been reported to be overexpressed in metastatic neuroendocrine prostate cancer (NEPC) and CRPC and is associated with poor clinical outcomes [151]. CBX2 depletion abolished cell viability and induced caspase-3-mediated apoptosis, suggesting that CBX2 may be a novel therapeutic target for advanced prostate cancer [152]. RNF2, the catalytic subunit of PRC1, is highly expressed in many different types of cancer. Studies have shown that the expression of RNF2 in prostate cancer tissues is higher than that in benign prostatic hyperplasia (BPH) tissues, and the knockdown of RNF2 in prostate cancer cells leads to cell cycle arrest, increased apoptosis, and the inhibition of cell proliferation and limits the tumor growth of xenograft models [153]. Therefore, it is necessary to introduce PRC1 as a novel biomarker and therapeutic target into the clinical treatment of prostate cancer.

### 4.10. PIKfyve

PIKfyve, a recently explored lipid kinase, has been shown to correlate with tumor activity and immune checkpoint blockades [154]. PIKfyve inhibition disrupts Toll-like receptors (TLRs) and cytokine signaling, such as IL12/IL13 signaling pathways [155,156]. Mice with the PIKfyve^−/−^ genotype show massive macrophage activation and inflammation [157]. By using the drug ESK981, a multiple-tyrosine kinase inhibitor which mainly targets VEGFR-1 and 2 [158], researchers found that it inhibited the activity of PIKfyve as well as induced the propagation of CXCL10 in prostate cancer [159]. It was also demonstrated that the knockdown of PIKfyve in Myc-CaP prostate cancer cells had tumor-inhibitory effects in both immune-competent and deficient mice, but the antitumor properties were maximized in a competent immune environment. Through PIKfyve inhibition, autophagic flux is also blocked, which means more chemokines such as CXCL10 can persist and recruit T cells to the TME. Combination treatments of PIKfyve knockdown with anti-PD-1 therapy led to significant increases in complete tumor regression, further supporting the inhibitory role PIKfyve plays in immunosuppressive signaling. Phase II clinical trials have already begun to test the effectiveness of ESK981 on its own (NCT03456804) or in combination with nivolumab (NCT04159896).

## 5. Conclusions and Future Direction

Immunotherapies have transformed the field of immuno-oncology, enabling some cancer patients to achieve durable immune control of their tumors, but have not been as effective in prostate cancer patients. Sipuleucel-T and immune checkpoint inhibitors have only been shown to be effective in selected early-stage patients and patients with deficient MisMatch Repair/High levels of MicroSatellite Instability (dMMR/MSI-H) tumors, respectively. The insignificant outcome of immunotherapy for advanced prostate cancer may be due to the complex immunosuppressive TME and the existence of multiple tumor immune evasion mechanisms in prostate cancer. Combination therapies such as targeting specific mechanisms in the TME and employing multiple strategies or drug combinations to overcome these resistance mechanisms in prostate cancer are currently at the center of immuno-oncology research. These innovative approaches have the potential to transform the prostate cancer TME from “cold” to “hot”, ultimately refocusing and revolutionizing the treatment of prostate cancer.

## Figures and Tables

**Table 1:** Completed vaccine and immune checkpoint inhibitor clinical trials for CRPC.

Clinical Trial Number and Trial Phase		Description	Results (OS in Months; PSA in ng/mL)	Ref.
Sipuleucel-T (NCT00065442)	III	Active cellular (peripheral-blood mononuclear and antigen-presenting cells)	OS, 25.8 with Spiuleucel-T 21.7 with placebo; PSA, 51.7 with Sipuleucel-T 47.2 with placebo	[27]
PROSTVAC (EudraCT 2010-021196-85)	III	Recombinant vaccinia and fowlpox viruses containing transgenes for human PSA and 3 T-cell costimulatory molecules	OS, 23.1 with viral vectors 22.8 with placebo; PSA, 71.4 with viral vectors 82.6 with placebo	[33]
VANCE (NCT02390063)	I	Replication-deficient viruses targeting oncofetal self-antigen 5T4 (early-stage PCa)	PSA, >100% increase in PSA levels post-vaccination for 3 participants, with others showing <50% increase	[34]
KRM-20 (UMIN000011028)	II	KRM-20 is a 20 peptide mix that induces cytotoxic T-lymphocytes against 12 tumor-associated antigens	No significant difference in PSA response, but both human leukocyte antigen (HLA)-IgG and CTL responses increased in KRM-20 arm	[35]
Blood-derived dendritic cells (DCs) (NCT02692976)	IIa	Monotherapies or combinations of myeloid DCs and/or plasmacytoid DCs used to induce cytotoxic T cells (intranodal injection)	Radiographic progression-free survival (rPFS) found to be 18.8 months in those with functional antigen-specific T cells (*n* = 5), and 5.1 months in those without (*n* = 16)	[36]
Anti-RhoC (NCT03199872)	I/II	The RhoC protein has been correlated with advanced cancer cells and metastasis, so this trial tests a vaccine to inhibit its function	86% of patients had a significant T-cell response during vaccinations, and 90% during the follow-up (functional T effector memory cells were seen, but not Tregs)	[37]
PCD4989g Atezolizumab (NCT01375842)	I	Small-molecule atezolizumab (PD-L1 inhibitor) with previous treatment using Sipuleucel-T or enzalutamide	PSA, 8.6% response, OS, 14.7 months, overall limited efficacy, so combination approach may be needed	[38]
IMbassador250 Atezolizumab (NCT03016312)	III	Small-molecule atezolizumab (PD-L1 inhibitor) with previous treatment using abiraterone; concurrent with enzalutamide for both arms	Stopped early because patients were at risk of immune-mediated adverse events; OS, 15.2 months for atezolizumab + enzalutamide vs. 16.6 months for enzalutamide only	[39]
KEYNOTE-199 Pembrolizumab (NCT02787005)	II	Monoclonal antibody pembrolizumab (PD-1 inhibitor) with previous treatment using docetaxel or enzalutamide	PSA, <10% response, ORR, <5%, rPFS, 2.1, 2.1, and 3.7 months for 3 cohorts (Cohort 1: PD-L1-positive; Cohort 2: PD-L1-negative; Cohort 3: bone-predominant disease, regardless of PD-L1 expression)	[40]
STARVE-PC Ipilimumab/Nivolumab (NCT02601014)	II	Ipilimumab (anti-CTLA4 monoclonal antibody), nivolumab (PD1 inhibitor), some concurrent treatment with nivolumab (all with enzalutamide)	Lower alkaline phosphatase levels in a subset of patients treated with immune blockade; did not meet primary endpoint	[41]
CheckMate650 Ipilimumab (NCT02985957)	II	Ipilimumab (CTLA4 inhibitor), nivolumab (PD1 inhibitor); one subset treated with cabazitaxe. Concurrent with nivolumab and higher dose ipilimumab	OS, 15.2 months in post-chemo cohort and 19 months in pre-chemo; ORR, 10% in post-chemo cohort and 25% in pre-chemo	[42]
MDX-010 Ipilimumab (NCT00323882)	I/II	Ipilimumab (CTLA4 inhibitor), dose-escalation treatments of ipilimumab combined with radiotherapy	PSA, 8 patients had PSA decline >50% and 1 had a complete response; high dose of 10 mg/kg ipilimumab showed a manageable safety profile	[43]

**Table 2:** Ongoing clinical trials utilizing immune checkpoint inhibitors in CRPC patients.

Trial Name and Trial Phase		Treatment(s)	Purpose and Expected Completion Date	
CHOMP (NCT04104893)	II	Pembrolizumab (PD-1 inhibitor)	To evaluate the activity and efficacy of pembrolizumab in mismatch repair deficiency (dMMR) and CDK12 biallelic inactivation mCPRC patients	3/2023
PERSEUS1 (NCT03506997)	II	Pembrolizumab (PD-1 inhibitor)	To evaluate the efficacy of pembrolizumab. To determine PD-1 and PD-L1, Treg infiltration, CD3, CD8, and lymphocyte infiltration	9/2025
NCT03406858	II	Pembrolizumab (PD-1 inhibitor), HER2Bi-armed activated T cells	To test if the combination of the HER2Bi-armed T cells and pembrolizumab is better at treating mCRPC patients	12/2021 (Active)
INSPIRE (NCT04717154)	II	Ipilimumab (CTLA4 inhibitor), nivolumab (PD1 inhibitor)	To evaluate the effects of 4 cycles of combination treatments (ipilimumab and nivolumab), followed by monotherapy nivolumab in participants with mCPPC	6/2025
IMPACT (NCT03570619)	II	Ipilimumab (CTLA4 inhibitor), nivolumab (PD1 inhibitor)	To evaluate the efficacy of combo treatment in patients with mCRPC and CDK12 mutations	5/2023
NCT03456804	II	ESK981 (Pan-VEGFR/TIE2 tyrosine kinase inhibitor and PIKfyve lipid kinase inhibitor)	To study the side effects and how well ESK981 works in treating patients with mCRPC	10/2022
NCT03792841	I	Acapatamab (bispecific T-cell engager), pembrolizumab (PD-1 inhibitor)	To determine the max tolerated dose of Acapatamab (a half-life extended (HLE) bispecific T-cell engager (BiTE^®^) construct) alone and in combination with pembrolizumab	6/2025
NCT05293496	I	MGC018 (CD276 inhibitor), lorigerlimab (dual PD-1 × CTLA-4 inhibitors)	To determine the safety and efficacy of MGC018 + lorigerlimab combo treatment	3/2025
NCT05177770	II	SRF617 (CD39 inhibitor), etrumadenant (dual A2aR/A2bR antagonist), zimberelimab (PD-1 inhibitor)	To evaluate the safety and efficacy of SRF617 in combination with etrumadenant and zimberelimab	11/2023
IceCAP (NCT03673787)	I/II	Ipatasertib (AKT inhibitor), atezolizumab (PD-L1 inhibitor)	Proof of concept for the combination of ipatasertib and atezolizumab acting on PI3K hyperactivated tumors	11/2023
NCT03061539	II	Nivolumab (PD1 inhibitor), ipilimumab (CTLA4 inhibitor)	To evaluate the efficacy of PD-1 inhibitor in combination with CTLA4 inhibitor	7/2025
NCT02933255	I/II	PROSTVAC-V/F (vaccine), nivolumab (PD1 inhibitor)	To evaluate the combination therapy of PROSTVAC and nivolumab for safety and effectiveness	8/2022
Rad2Nivo (NCT04109729)	Ib/II	Nivolumab (PD1 inhibitor), radium-223 (radioactive isotope)	To assess the safety of this combination treatment, then expand into a phase II cohort	4/2025
NCT04159896	II	ESK981 (multi-tyrosine kinase inhibitors), nivolumab (PD1 inhibitor)	To evaluate the safety and efficacy of these drugs in combination (ESK981 = pan-VEGFR/TIE2 tyrosine kinase inhibitor)	3/2022 (Active)
NCT03651271	II	Nivolumab (PD1 inhibitor), ipilimumab (CTLA4 inhibitor)	To evaluate treatment outcomes for patients with low vs. high levels of CD8 cells in tumor biopsy in monotherapies of nivolumab or combo	5/2023
CheckMate 7DXNCT04100018	III	Nivolumab (PD1 inhibitor), prednisone, docetaxel	To assess the safety and efficacy of nivolumab + docetaxel in comparison to placebo + docetaxel	8/2027
NCT05169684	II	BMS986218 (CTLA4 inhibitor), docetaxel, nivolumab (PD1 inhibitor)	To assess the safety and efficacy of BMS986218 in different combos with nivolumab and docetaxel	2/2026
PORTER (NCT03835533)	I	NKTR-214 (CD122-preferential IL2 pathway agonist), nivolumab (PD1 inhibitor), SBRT (radiation), CDX-301 (FLT3 ligand, a dendritic cell mobilizer), INO-5151 (combination of DNA plasmids encoding IL-12 and PSA/PSMA)	To evaluate the safety and efficacy of immunotherapy combinations. To explore immune biomarker response in prostate cancer after treatment with different combinations	3/2023
STELLAR-001(NCT03845166)	I	XL092 (tyrosine kinase inhibitor that targets VEGF receptors, c-Met), atezolizumab (PD-L1 inhibitor), avelumab (PD-L1 inhibitor)	To evaluate the safety, tolerability, pharmacokinetics (PK), preliminary antitumor activity by XL092 as a monotherapy or in combination with other PD-L1 inhibitors	11/2024
